# Update: Noncongenital Zika Virus Disease Cases — 50 U.S. States and the District of Columbia, 2016

**DOI:** 10.15585/mmwr.mm6709a1

**Published:** 2018-03-09

**Authors:** Victoria Hall, William L. Walker, Nicole P. Lindsey, Jennifer A. Lehman, Jonathan Kolsin, Kimberly Landry, Ingrid B. Rabe, Susan L. Hills, Marc Fischer, J. Erin Staples, Carolyn V. Gould, Stacey W. Martin

**Affiliations:** ^1^Epidemic Intelligence Service, CDC; ^2^Arboviral Diseases Branch, Division of Vector-Borne Diseases, National Center for Emerging and Zoonotic Infectious Diseases, CDC.

Zika virus is a flavivirus primarily transmitted to humans by *Aedes aegypti* mosquitoes (*1*). Zika virus infections also have been documented through intrauterine transmission resulting in congenital infection; intrapartum transmission from a viremic mother to her newborn; sexual transmission; blood transfusion; and laboratory exposure (*1*–*3*). Most Zika virus infections are asymptomatic or result in mild clinical illness, characterized by acute onset of fever, maculopapular rash, arthralgia, or nonpurulent conjunctivitis; Guillain-Barré syndrome, meningoencephalitis, and severe thrombocytopenia rarely have been associated with Zika virus infection (*1*). However, congenital Zika virus infection can result in fetal loss, microcephaly, and other birth defects (*1*,*2*). In 2016, a total of 5,168 noncongenital Zika virus disease cases were reported from U.S. states and the District of Columbia. Most cases (4,897, 95%) were in travelers returning from Zika virus-affected areas. A total of 224 (4%) cases were acquired through presumed local mosquitoborne transmission, and 47 (1%) were acquired by other routes. It is important that providers in the United States continue to test symptomatic patients who live in or recently traveled to areas with ongoing Zika virus transmission or had unprotected sex with someone who lives in or traveled to those areas. All pregnant women and their partners should take measures to prevent Zika virus infection during pregnancy. A list of affected areas and specific recommendations on how to prevent Zika virus infection during pregnancy are available at https://www.cdc.gov/pregnancy/zika/protect-yourself.html.

Before 2015, local transmission of Zika virus had been reported in Africa, Southeast Asia, and the Pacific Islands (*1*). In 2015, local mosquitoborne transmission of Zika virus was first identified in Brazil and subsequently spread throughout the Region of the Americas. To date, 48 countries and territories in the Americas have had confirmed mosquitoborne transmission of Zika virus (*4*). In the United States, Zika virus disease and congenital Zika virus infection became nationally notifiable conditions in February 2016, when the Council of State and Territorial Epidemiologists (CSTE) approved interim case definitions (*5*). In June 2016, CSTE approved revisions to the laboratory criteria and the addition of asymptomatic Zika virus infections to the case definitions (*6*). States were asked to reclassify their Zika virus disease cases according to the revised definitions. This report describes confirmed and probable cases of noncongenital Zika virus disease with illness onset during 2016, reported from U.S. states and the District of Columbia to ArboNET, the national arboviral surveillance system. Cases were classified as confirmed or probable according to clinical, epidemiologic, and laboratory-testing criteria. Asymptomatic noncongenital Zika virus infections and all congenital Zika virus infections were excluded from this summary. More information on reported congenital infections is available at https://www.cdc.gov/zika/reporting/pregnancy-outcomes.html.

A total of 5,168 noncongenital Zika virus disease cases with symptom onset during January 1–December 31, 2016, were reported to ArboNET (Figure 1). The number of reported cases peaked in July and declined rapidly after August. Although cases were reported from 49 states and the District of Columbia, approximately half (48%) were reported from three states (Florida [1,107; 21%], New York [1,002; 19%], and California [421; 8%]) (Figure 2).

**FIGURE 1:**
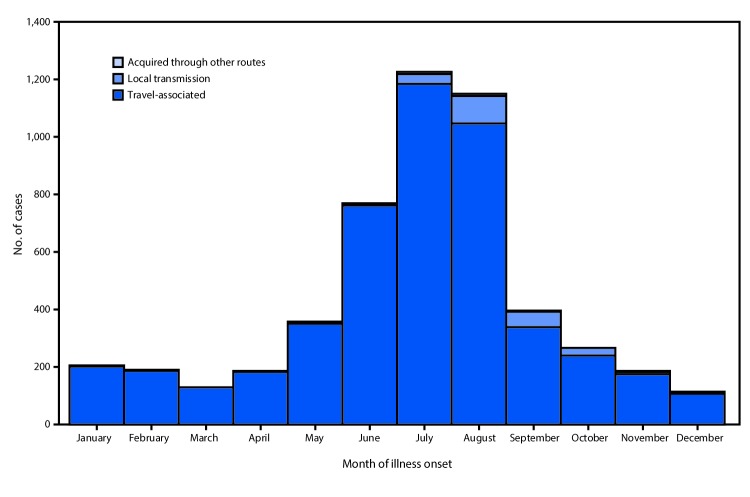
Noncongenital Zika virus disease cases (N = 5,168),* by month of illness onset — 50 U.S. states and the District of Columbia, January 1–December 31, 2016 * Other routes include 47 reported cases that were transmitted through sexual contact (45), laboratory exposure (one), and person-to-person through an unknown route (one).

**FIGURE 2:**
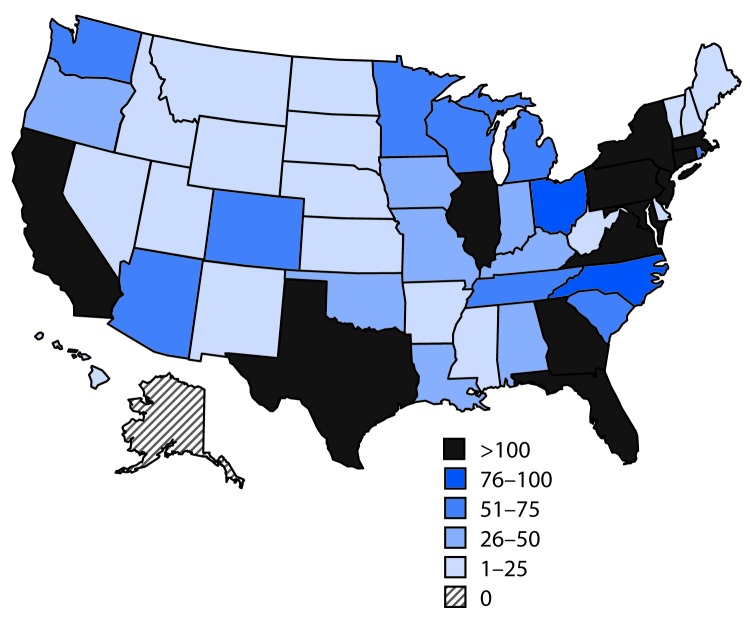
Number of confirmed and probable Zika virus disease cases, by state of residence — 50 U.S. states and the District of Columbia, January 1–December 31, 2016

The median age of patients with Zika virus disease was 37 years (range = 10 months–89 years), with 4,118 (80%) aged 20–59 years (Table). Overall, 3,310 (64%) cases occurred in females, and a higher proportion of female patients (24%) were aged 20–29 years compared with male patients (16%). Among the 3,310 Zika virus disease cases that occurred in females, 469 (14%) were in pregnant women.

**TABLE:** Characteristics of confirmed and probable noncongenital cases of Zika virus disease — 50 U.S. states and the District of Columbia, January 1–December 31, 2016

Characteristic	No. (%)		
	Female (n = 3,310)	Male (n = 1,858)	Total (N = 5,168)
**Age group (yrs)**			
0–9	60 (2)	33 (2)	93 (2)
10–19	249 (8)	155 (8)	404 (8)
20–29	794 (24)	298 (16)	1,092 (21)
30–39	771 (24)	440 (24)	1,211 (23)
40–49	600 (18)	387 (21)	987 (19)
50–59	499 (15)	329 (18)	828 (16)
≥60	335 (10)	214 (12)	549 (11)
Unknown	2 (<1)	2 (<1)	4 (<1)
**Transmission mode**			
Travel-associated	3,163 (96)	1,734 (93)	4,897 (95)
Local mosquitoborne	103 (3)	121 (7)	224 (4)
Other*	44 (1)	3 (<1)	47 (1)
**Clinical outcome**			
Hospitalized	111 (3)	42 (2)	153 (3)
Died	0 (0)	1 (<1)	1 (<1)

* Includes sexual transmission (45), laboratory exposure (one), and person-to-person through an unknown route (one).

Guillain-Barré syndrome was reported in 15 (0.3%) cases; the median age of these patients was 61 years (range = 27–81 years). Overall, 153 (3%) patients were hospitalized (Table); the median age of hospitalized patients was 41 years (range = 1–89 years). Among the 111 females hospitalized with Zika virus disease, 25 (23%) were pregnant. One hospitalized male patient died (*7*).

Among all 5,168 reported cases, 4,897 (95%) occurred in travelers returning from areas with Zika virus transmission (Table). The most common travel destination among the 3,891 (79%) cases for which this information was available was the Caribbean (2,389; 61%), followed by Central America (766; 20%), North America (521; 13%), South America (195; 5%), and Southeast Asia and the Pacific Islands (20; <1%).

Presumed local mosquitoborne transmission was the source of infection for 224 (4%) Zika virus disease patients, including 218 in Florida and six in Texas (Figure 1). The first autochthonous, mosquitoborne cases in the continental United States occurred in Florida in June 2016; local transmission peaked in August and then sharply declined. The patients with locally transmitted disease in Texas all had reported onset in November and December. The median age of patients with local mosquitoborne disease was 37 years (range = 7–81 years) and 103 (46%) were female.

Forty-seven (1%) cases were acquired through other routes, including sexual transmission (45), laboratory transmission (one), and person-to-person through an unknown route (one) (Table). The median age of patients with reported sexually transmitted Zika virus disease was 29 years (range = 18–61 years) and 43 (96%) were female.

## Discussion

From 2007 to 2014, only 14 travel-associated cases of Zika virus disease were recognized in the United States (*1*,*8*). Following the introduction and spread of Zika virus in the Americas in 2015, the number of travel-associated cases in U.S. states increased, with 4,897 cases reported in 2016. The number of reported travel-associated cases in the United States peaked during July 2016 and declined during the second half of the year. An additional 224 cases attributable to local mosquitoborne transmission were reported during 2016 and were geographically limited to small areas in Florida and Texas.

Similar to the U.S. experience with both dengue and chikungunya, also transmitted by *Aedes aegypti*, most Zika virus disease cases occurred among travelers recently returning from locations outside the continental United States (*9*). The geographic and seasonal pattern of reported local mosquitoborne Zika virus transmission in the United States was also similar to prior local transmission of chikungunya and dengue viruses. Despite the presence of *Aedes aegypti* in multiple states, other environmental conditions (e.g., use of air conditioning and window screens, temperate climate, lower human population density, and reduced mosquito habitat) likely limited the transmission risk in U.S. states. If Zika virus disease trends continue to follow these historical mosquitoborne disease patterns, cases among travelers will continue to occur, but at lower levels, and limited local transmission with sporadic cases or clusters is possible. During the first 8 months of 2017, the number of reported cases (331) was markedly lower than the number reported during the same time frame in 2016 (4,205). Current data are available at https://www.cdc.gov/zika/reporting/case-counts.html.

The demographic characteristics of Zika virus disease cases reported by U.S. states in 2016 are similar to those described in an earlier report summarizing data from the first 6 months of 2016 (*7*). Overall, 64% of cases reported in 2016 occurred in females, and a higher percentage of the female patients were aged 20–29 years. These findings are likely driven by increased testing of women of childbearing age because of concerns about possible congenital infection. This hypothesis is supported by the local mosquitoborne disease data where more active surveillance and testing was performed and cases were more equally distributed between males and females. Other factors also might have contributed to the higher proportion of reported female travel-associated cases (e.g., differential health care–seeking, mosquito exposure, or sexual transmission).

The findings in this report are subject to at least three limitations. First, the case numbers are likely an underestimate because most cases are mild, which might result in persons not seeking health care or clinicians not ordering diagnostic tests. Second, because ArboNET cases from early 2016 had to be reclassified to reflect the June 2016 case definition changes, some cases might have not been correctly recategorized. Finally, a number of different diagnostic tests are in use and might vary in diagnostic accuracy. For this reason, false positive or false negative test results might result in cases being missed or incorrectly diagnosed and reported.

This report did not include data from the U.S. territories; Puerto Rico, U.S. Virgin Islands and American Samoa experienced large outbreaks of Zika virus in 2016 that were the result of local mosquitoborne transmission. Because the epidemiology of Zika virus was very different in U.S. states as compared to U.S. territories, this report focuses on cases reported from U.S. states only.

CDC continues to recommend that health care providers test patients with a clinically compatible illness who live in or recently traveled to areas with ongoing Zika virus transmission or had unprotected sex with someone who lives in or traveled to those areas (https://www.cdc.gov/zika/hc-providers/testing-guidance.html). In July 2017, new guidance was released for providers caring for pregnant women with possible Zika virus exposure to reflect the lower positive predictive value of diagnostic testing in the setting of decreasing prevalence of disease and the difficulty in determining the timing of infection based on serologic testing as the outbreak continued (*2*). All pregnant women should be asked about possible Zika virus exposure before and during the pregnancy at every prenatal care visit to guide appropriate diagnostic testing and clinical care. Interim guidance for the evaluation of infants with possible congenital Zika virus exposure has been published (*10*). CDC continues to recommend that pregnant women avoid travel to areas with risk for Zika virus transmission, and all pregnant women and their partners should take measures to prevent Zika virus infection during pregnancy.

Timely identification and investigation of cases, especially in areas with *Aedes aegypti* mosquitoes, will reduce the risk for local mosquitoborne transmission in the continental United States. Although the risk for travel-associated Zika virus disease appears to be decreasing, all persons should continue to take precautions when traveling to areas with a risk for Zika virus transmission, including using strategies to prevent mosquito bites and sexual transmission. Additional information is available at https://www.cdc.gov/zika/.

**Summary**

**What is already known about this topic?**

Zika virus disease is an arboviral disease usually causing mild illness; however, congenital infection is associated with microcephaly and other birth defects. Although most cases in residents of U.S. states were travel-associated, local transmission has been reported.

**What is added by this report?**

In 2016, a total of 5,168 confirmed or probable cases of noncongenital Zika virus disease with symptom onset during January 1–December 31, 2016, were reported to ArboNET from U.S. states and the District of Columbia. Most (95%) cases were travel-associated. Locally acquired disease accounted for 4% of cases, with transmission occurring in Florida (218) and Texas (six). Forty-seven cases (1%) were acquired through other routes, including sexual transmission (45), laboratory transmission (one), and person-to-person through an unknown route (one).

**What are the implications for public health practice?**

CDC recommends that health care providers continue to test patients with a clinically compatible illness who live in or recently traveled to areas with ongoing Zika virus transmission or had unprotected sex with someone who lives in or traveled to those areas (https://www.cdc.gov/zika/hc-providers/testing-guidance.html). Although the risk for travel-associated Zika virus disease appears to be decreasing, it is important that persons traveling to areas with a risk for Zika virus transmission continue to take precautions, including using strategies to prevent mosquito bites and sexual transmission.
